# Experimental approaches for addressing fundamental biological questions in living, functioning cells with single molecule precision

**DOI:** 10.1098/rsob.120090

**Published:** 2012-06

**Authors:** Tchern Lenn, Mark C. Leake

**Affiliations:** 1 Lawrence Berkeley National Laboratory, Physical Biosciences Division, 1 Cyclotron Road, Berkeley, CA 94720, USA; 2 Clarendon Laboratory, Department of Physics, Oxford University, Parks Road, Oxford OX1 3PU, UK

**Keywords:** single molecule, *in vivo*, microscopy, super-resolution, fluorescence imaging, fluorescent protein

## Abstract

In recent years, single molecule experimentation has allowed researchers to observe biological processes at the sensitivity level of single molecules in actual functioning, living cells, thereby allowing us to observe the molecular basis of the key mechanistic processes in question in a very direct way, rather than inferring these from ensemble average data gained from traditional molecular and biochemical techniques. In this short review, we demonstrate the impact that the application of single molecule bioscience experimentation has had on our understanding of various cellular systems and processes, and the potential that this approach has for the future to really address very challenging and fundamental questions in the life sciences.

## 2. Introduction: the single biological molecule approach

In terms of invoking a technology in successfully addressing outstanding questions in biology, barring some rare exceptions, the single molecule experiments that have most pushed the field forward have involved fluorescence imaging in some form. Fluorescence microscopy can generate exceptional measurement contrast for the detection of tiny signals emitted from single molecule fluorescent tags in the milieu of a living cell, either in the form of fluorescent organic dyes or, as is increasingly more common in live-cell work, by the use of genetically encoded fluorescent proteins. Fluorescence imaging manages to achieve this high detection contrast in a way that is in general relatively minimally perturbing to the native biological context. It is the least invasive technique that allows robust detection of single molecules in a near physiological environment, making it quite simply the clear biophysical technique of choice. Modern single molecule fluorescence imaging now involves a suite of novel technologies that has been a key tool in a broader context towards addressing fundamental life-science questions, most especially in cell biology. This has essentially spawned a new science of *single molecule cell biology* or *single molecule cellular biophysics*, characterized by the use of cutting-edge tools of biophysics applied at the single cell level and at the single molecule level.

This approach is concerned with biology on the scale of just one cell, predicated on the understanding that a given sub-cellular component is, in reality, a population of individual molecules within a wider cellular context. Furthermore, the population *structure*, that is not only the surrounding environment in which a single molecule is found but also the architecture of its network of interactions with other molecules in the cell, determines its real function in practice within the functioning biological system. This population is potentially influenced by highly complex feedback over multiple length and time scales, which in principle may span several biological systems that classical bulk biochemical observations may deem as being ostensibly unrelated. In other words, in order to thoroughly understand the real workings of any given single biological process, it is often necessary to take into account the actions of other systems in the cell. There has therefore been a move towards performing single molecule experiments either in the native cell or in a complex test tube level *in vitro* environment, which involves far more physiologically relevant components than were present in the early first generation of single biomolecule experiments—these latter approaches were exceptional in their ingenuity but limited in being performed essentially on isolated molecules divorced from their true cellular context.

Traditional molecular and biochemical approaches to cell biology have been concerned with the mean average properties of a large population of molecules. For example, a single microlitre of water contains approximately 10^19^ molecules. These ‘bulk’ methods are predicated on the assumption that the mean value from the population is an appropriate value to represent the whole population of molecules. However, biomolecules of a single given type in general will exist in a variety of energetic and conformational states. Essentially, in terms of understanding biological molecules as existing on a free energy *landscape*, we would observe most to be present in a multitude of meta-stable states. In other words, there is an intrinsic energetic *instability* to single molecules that is often essential to their biological function, and this is manifest as a typically heterogeneous distribution of a given molecular property. Thus there is, in all but the very exceptional cases, significant heterogeneity in behaviour at the molecular level. This can be manifest in terms of both spatial and temporal properties—that is, in general, molecules within a population lack *synchronicity*, barring some well-studied exceptions such as some types of molecules expressed in muscle tissue, in terms of both the spatial locations and the timing of when they change to a different energetic state. In a nutshell, this means that it may be non-trivial to reliably associate a mean ensemble value as being truly representative of the behaviour of many molecules. Instead, what is needed is the ability to sample single molecules *one-by-one* to actually build up a characteristic *distribution* of molecular properties.

The single molecule approach is concerned with measuring the heterogeneity within a population by direct measurement of biochemical and physical properties of individual molecules. This method therefore aims to uncover the underlying principles and mechanisms that govern the function of systems in cell biology by observing the population structure of molecular components therein, one molecule at a time. Single molecule experiments therefore in general make observations and measurements on smaller spatial, temporal and numerical scales than bulk ensemble experiments, but with the caveat that many such observations often need to be made in order to properly construct the underlying distribution of molecular properties required. This approach is typically exceptionally data-rich and computationally demanding, requiring specialist instrumentation and highly interdisciplinary co-operation of experts in the biological, physical, chemical, engineering, mathematical and computational sciences in order to draw good conclusions from the data. Single molecule observations are highly precise and sensitive measurements, requiring confidence in the measurement apparatus for data acquisition and the accurate extraction of relevant information in the various analysis stages.

The experimental methodologies used for single molecule studies have been reviewed extensively elsewhere (see Lord *et al.* [1], which also gives a good historical account of the single molecule approach, and Harriman & Leake [2], which gives a more physical science perspective), and the reader is strongly encouraged to look through these reviews to get a background understanding of the techniques of the field. Here, however, we provide a *bio-centric* overview of the *utility* of the methodology—namely, what range of real, biological questions can modern single molecule cellular biophysics techniques actually address, and ultimately hope to demonstrate the enormous impact of live-cell single molecule imaging on ‘traditional’ biological disciplines.

## 3. Single molecule biochemistry: understanding protein structures and biochemical processes *one molecule at a time*

The traditional approach to studying the structure and biochemistry of proteins, which still provides enormously useful and complementary information to single molecule approaches, is to isolate the protein of interest for biochemical assays and thereby infer how that protein works in the cell from which it was taken. While this approach often does yield significant models with great predictive power, models thus generated must be validated *in vivo* to be proved to be representative as to what really happens physiologically. In the example of the DNA replication machinery, earlier *in vitro* studies on the model organism *Escherichia coli*, by traditional biochemical approaches, lead to conflicting models when compared with the results of more recent *in vitro* and *in vivo* structure and function studies. The *E. coli* replication factory is called the *replisome*; it is a multi-subunit machine consisting of more than 10 different protein components that function in a highly coordinated way, and is a good example of a *mesoscale* molecular structure in the cell, which can be understood best by using advanced single molecule techniques.

**Figure 1.**
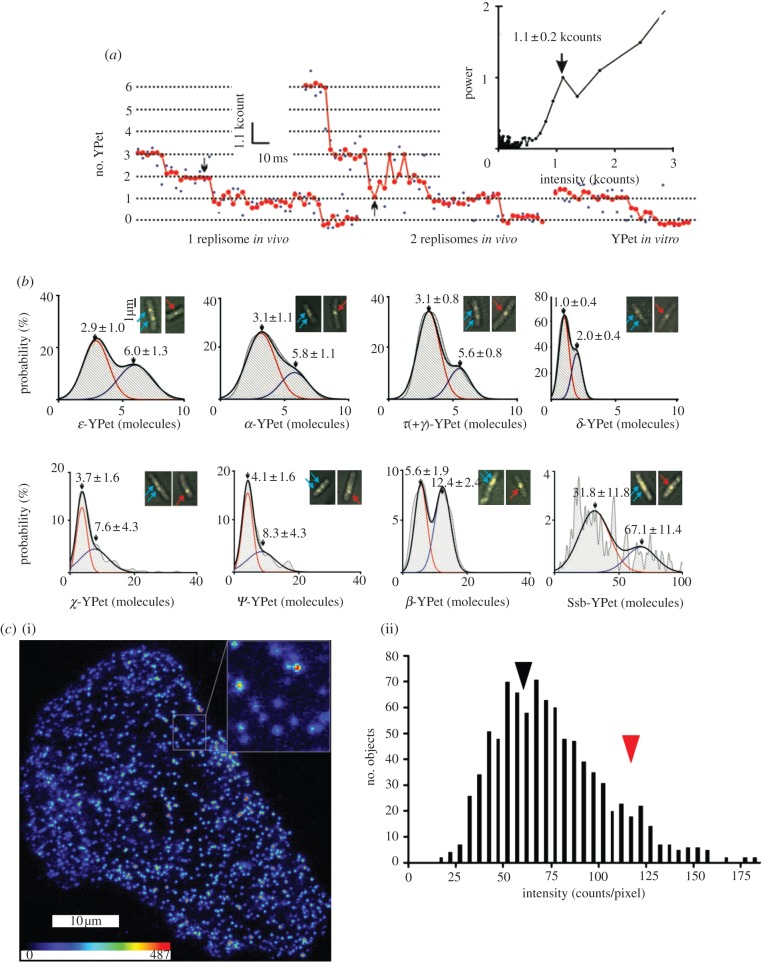
Stoichiometry of proteins *in vivo*. (*a*,*b*) Stoichiometries of the components of *E. coli* replisomes as determined by step-wise photobleaching of diffraction-limited spots in slimfield fluorescence images (*n* = 27–51 cells in each dataset), adapted from [7]. (*a*) In this approach, bleaching step size is determined by Fourier spectral analysis (upper right) of the pair-wise difference distribution function of bleaching traces (raw data in blue, filtering result in red) filtered with a Chung–Kennedy edge preserving filter, and the number of fluorescent molecules was determined by the quotient of the background corrected initial intensity and the bleaching step [7–12]. (*b*) Shown are two-Gaussian fits (black) with contributing single-Gaussian curves (red and blue) and mean ± s.d. of Gaussian peaks. Insets show examples of overlaid brightfield (grey) and single 3 ms fluorescence images (yellow) for each; arrows indicate foci in cells containing two (cyan) and one (red) replisome. Two populations can be seen in each KDE corresponding to data from cells with single and double foci. (*c*) Co-existence of monomeric and dimeric M1 muscarinic receptors in CHO cells, adapted from [13]. (i) pseudo-coloured TIRFM image of Cy3B-telepenzine labelled receptors. (ii) Histogram of the intensity of 911 objects identified in the single video frame shown above. The shape of the distribution is due to the presence of two populations, attributed to receptor dimers (red arrowhead, intensity approx. 120 counts per pixel) and monomers (black arrowhead, approx. 60 counts per pixel). The spread of the data arises from a combination of photon noise and intensity variation between spots that are located at different regions within the specimen.

Replisomes consist of heterotrimeric DNA polymerases (Pol III) attached via multi-subunit clamp-loaders to a single hexameric helicase (DnaB), which unwinds and separates double-stranded DNA at the replication fork. Traditionally, the *E. coli* replisome was thought to contain two copies of the polymerase enzyme [3,4], one on each DNA strand in the replication fork, and also because two-polymerase replisomes were known to be able to synthesize double-stranded DNA *in vitro*, albeit at substantially impaired rates compared with the native cellular systems. More recently, however, observations that three-polymerase replisomes spontaneously formed from the components *in vitro* and were functional for DNA replication suggested that the number of polymerases in the replisome *in vivo* might instead be three [5,6]. This controversy is due in part to an ambiguity from *in vitro* data over the stoichiometry of the τ subunit of the clamp loader, which oligomerizes Pol III in a 1 : 1 stoichiometry, as it is present in isolated replisomes together with a frame-shifted truncation of the **τ** subunit assigned **γ**, which does not bind Pol III and is difficult to distinguish from **τ** biochemically. By using the so-called *step-wise photobleaching* in a *slimfield* illumination regime, Reyes-Lamothe *et al.* [7] were able to determine the *in vivo* stoichiometry of multiple components of the *E. coli* DNA replication machinery (figure 1*a,b*). A number of features of this seminal study illustrate the uniqueness and utility of single molecule fluorescence microscopy, and also some of the dangers of misinterpreting results if care is not taken to understand the fine details of single molecule analysis, and we will use it as a thoroughly worked case study here in our review.

First of all, the motivation to pursue a single molecule imaging approach for this question was the clear case where direct, *in vivo* observation of the stoichiometry of the functioning protein complex in question would resolve conflicting models. This was also a case where traditional fluorescence microscopy approaches, in terms of both data collection and analysis, suited for reliably locating and determining relative abundances of a large (typically greater than 1000) number of molecules, lacked the sensitivity to reliably count the few fluorophores (less than 100) expected to feature in such an experiment. In actual fact, the apparatus required for *in vivo* single molecule imaging is not necessarily different from that required for traditional fluorescence imaging.

However, the devices involved are required to perform at or near the limits of what is theoretically and actually possible to reliably collect single molecule data; single molecule fluorescence imaging tends to be carried out by expert biophysicists on custom-built microscopes with lasers as excitation sources and high-performance electron-multiplying CCD cameras as detectors. Such equipment is also available in most well-endowed bio-imaging facilities around the world, but is often not used at anything approaching the real sensitivity limits. This has resulted in a tragic waste of finite research funds; such equipment has been used to generate ‘pretty’ live-cell images that have not been interrogated robustly for molecular-level quantification. This can be easily resolved by forethought in establishing much better and more transparent collaboration between ‘card-carrying’ life scientists and the single molecule biophysicts who are expert at extracting the molecular-level signatures from live-cell data.

For data collection in this DNA replication study, each replisome component was separately labelled in different bacterial cell strains of *E. coli* using the yellow fluorescent protein YPet, which was imaged using a novel method called slimfield. In slimfield illumination, the excitation laser light is squeezed just into the vicinity of a single cell, and this can be used to generate very high excitation intensities, which overcome much of the normal imaging noise present when acquiring very fast images at the millisecond time scale [8].

In many examples of earlier single molecule live-cell studies of components integrated into the cell membrane, the well-characterized technique of total internal reflection fluorescence (TIRF) microscopy could be used to generate substantial imaging contrast over the autofluorescence background from the cytoplasm, using imaging integration times of several tens of milliseconds, or the so-called *video-rate*. However, a cell's replication machinery is located *inside* the cell as opposed to that on its surface, hence the need for novel non-TIRF imaging methodology. Components that are expressed in the cytoplasm move much faster than those in the cell membrane, owing to the viscosity being smaller by a factor of approximately 100–1000, which means that the imaging integration time needs to be on the millisecond level to observe components unblurred, but at such small exposure times the background noise from the camera detector is normally much higher than the signal generated when using typical video-rate imaging, even in high contrast approaches such as TIRF. Rather, the slimfield illumination used permitted such rapid imaging in giving the YPet fluorescent protein reporter molecules used on the different replisome components in effect a signal ‘boost’ over the camera noise, allowing the investigators to record fluorescence intensity photobleaching traces from localized diffraction-limited spots of fluorescence observed in each single bacterial cell, with enough data points between bleaching events to generate a step-wise intensity profile as a function of time over which the sample is illuminated.

This study showed that each replisome is likely to contain three Pol III polymerases *in vivo*. Over-zealous criticism of this study by McHenry [14] demonstrates how easily single molecule data can be misunderstood by life scientists with little understanding of the field, and shows the care needed in interpretation of single molecule data, and the need by scientists new to the field to try to at least get to grips with the core details of the single molecule analyses involved, which are unfortunately, in most modern research publications, described as a bundle in electronic supplementary material for space considerations, making the process of following such fine detail difficult for the reader.

Contrary to McHenry's apparent understanding of the data here, the conclusion that the *E. coli* replisome contains three polymerases *in vivo* is not based on an observed ‘triple-step’ photobleaching. Rather, as detailed in supporting online information, step-wise bleaching in combination with data-filtering and Fourier spectral analysis was used to estimate the average fluorescence intensity of a single YPet molecule, the value of which was determined to be reasonable in this study by two independent methods—(i) recording the fluorescence intensity of immobilized single YPet molecules and (ii) the peak position of the smallest intensity class in a distribution generated from binned raw intensity measurements. The quotient of the background-corrected initial intensity of auto-detected spots and the fluorescence intensity of a single YPet molecule was then taken to be the measured number of YPet molecules in the diffraction-limited spots. This ratiometric approach was favoured over ‘step counting’ by direct inspection of photobleaching time series traces owing to the intrinsic subjectivity and uncertainty associated with identifying single steps in raw photobleaching traces, even when using automated step-detection algorithms. Camera noise, cell-to-cell variation in the autofluorescence background and the stochastic nature of photobleaching, which means that multiple fluorophores may simultaneously bleach between time points, all contribute to the uncertainty in defining a single step reliably for multiple traces from ‘serial-interrogation’ of the photobleach trace, using methods that apply running detection windows along the time axis of the intensity data.

The probability distribution obtained by this analysis for the number of YPet molecules present per fluorescent ‘spot’ in a single cell for a given fluorescently tagged replisome component was reported in the form of unbiased *kernel density estimations* (KDEs) as shown in figure 1. KDEs replaced the traditional histogram in this study, and are being used with increasing prevalence in single molecule analysis elsewhere. Essentially, each data point is replaced by a Gaussian function whose width corresponds approximately to the estimated experimental measurement error and whose area is normalized to unity, thus corresponding to a best guess for the expected sampling distribution for each measurement on the basis of the data that is available. Thus, the entire dataset is in effect convolved with a function that represents real measurement sampling error, and the resultant distribution no longer relies on subjective bias prevalent in the use of histograms, for example, on the width of the bins or precisely where the bin edges lie.

From these unbiased KDEs, it was clear that there was a spread of measured YPet molecules in the detected fluorescence spots for each *E. coli* strain analysed. Some of the error in the final estimation of stoichiometries is associated with the fact that the average fluorescence intensity of a single YPet molecule thus calculated may either be a marginal over or underestimate for a given spot. Other sources of error include the presence of a dark population of fluorophores—a consideration for all single molecule fluorescence imaging experiments which use fluorescent proteins—as well as natural variation in stoichiometry, because complexes dynamically turn over and assemble. The rate-limiting step for the generation of the ultimate photoactive ‘mature’ state of a fluorescent protein is an oxidation reaction, and it is possible by suitable genetic mutation to generate variants of fluorescent proteins that mature relatively quickly *in vivo*, or which at least can be well-characterized experimentally in terms of maturation time and the likely proportion of dark population.

McHenry's criticism that ‘No statistics were presented regarding the number of foci that yielded photobleaching profiles consistent with expectations’ is therefore erroneous in two senses: firstly in the misunderstanding that the KDEs shown were of detected step numbers directly from photobleaching traces, and secondly that the statistics are not presented, because the distributions exactly reflect the statistics of the number of ‘foci’, or spots, that possess all measured stoichiometries. McHenry does make a valid point in that the errors reported make the determination of stoichiometries uncertain, but this is true only in the sense that there is uncertainty associated with measurement of any particular parameter in a population.

The excellent bimodal fits for two-Gaussian distributions of the replisome components, **α**, **ε**, **δ**, **β** and **τ**, reflect the largely normal distribution of spot stoichiometries in two populations around two mean values. The fact that the population of spots contributing to the higher mean value in all cases came from cells where only one spot (instead of two) was visualized in combination with the observation that, for all cases shown, the higher mean value is approximately twice the lower mean value is convincing evidence that the mean value of the populations with lower stoichiometries is the true expected stoichiometry corresponding to a given tagged replisome component from a single replication fork.

In fact, the normal distributions compel the reader to reasonably expect that any given replisome will have three polymerases, as the data indicate single populations with a mean of approximately three copies of all polymerase subunits. Furthermore, the ability to resolve the separate populations of **δ** from cells with one or two detectable spots, which differ by just one molecule, should convince the reader that distinct populations of two-polymerase and three-polymerase replisomes should be detected, if indeed there is a population of two-polymerase replisomes in *E. coli.* In contrast to polymerase subunits, the non-normal distributions of **χ**, **ψ** and Ssb in particular lead to a conclusion that it is not reasonable to expect a fixed number of these molecules to be associated with a given replisome.

The variable stoichiometry observed for the Ssb component, a protein used to stabilize single-stranded DNA, is an example of information that is uniquely contributed by the single molecule approach, in stark contrast to bulk ensemble average measurements that could tell us nothing conclusive about the real shape and extent of the probability distribution for stoichiometry here. Analysis of the stoichiometry distribution of Ssb also indicated that a tetramer was the fundamental Ssb subunit, because the multiple peaks in the Ssb stoichiometry KDE were separated by approximately 4-mer intensity units, as determined by Fourier spectral analysis.

McHenry also suggests that the reported 30 per cent increase of **τ** stoichiometry in the absence of **γ** is consistent with a *τ*2γ1 model. However, we note that a *τ*2γ1 model would predict a 50 per cent increase in **τ** stoichiometry rather than the reported 30 per cent increase, which is actually consistent with a *τ*3γ1 model.

A more valid critique of the data presented by Reyes-Lamothe *et al.*, characteristic of many single molecule investigations, is the apparent paucity of data used to construct each distribution of single molecule properties, owing primarily to the non-trivial technical challenges of performing these experiments in live cells. For example, the fact that some of the KDEs were constructed from as few as 27 cells indicates that there were approximately 54 spots observed, and it is difficult for a reader to determine whether an apparent break in a bimodal pattern for the **χ**, **ψ** and Ssb components is due to under-sampling of these populations, or whether there was sufficient sampling to validate the conclusion that the other replisome components sampled belong to a single population. Improvements to such single molecule experiments could be made by the application of robust statistical tests throughout multiple stages of analysis. Nonetheless, the observations of Reyes-Lamothe *et al.* have subsequently been independently reproduced by Lia *et al.* [15], who used a stroboscopic illumination to capture turnover of Pol III and correlated this with fluctuations in Ssb concentration at the replication fork suggesting that the third polymerase is involved in lagging strand synthesis *in vivo.* The case for a three-polymerase replisome, first suggested *in vivo* by single molecule imaging, is further strengthened but the recent demonstration that three-polymerase replisomes have higher processivity and efficiency in lagging strand synthesis than two-polymerase replisomes *in vitro* [16], suggesting a function for the third polymerase *in vivo*.

Another good example of how molecular heterogeneity can be sampled *in vivo* using single molecule methods comes from the multiple oligomeric states of the protein TatA, the pore-forming component of the bacterial translocase system for folded proteins [17], which strongly support the model that TatA forms rings of varying sizes, dependent on other twin-arginine transport (Tat) proteins, which had been speculated by earlier *in vitro* biochemical data [9]. A further example of successfully sampled molecular-cellular heterogeneity comes from a study that looked at a component protein of the phage-shock protein response system in *E. coli*, PspA, which is known to be a bi-functional protein, as both an actuator and an effector of the system [10,18]. The two functions of PspA are predicted by *in vitro* biochemical methods and *in vivo* molecular genetics experiments to relate to variable oligomeric states of PspA. Although this study also potentially suffered from the criticism of under-sampling of cell data, live-cell single molecule imaging provided support of a model [19] where hexamers of PspA are related to the actuator function of PspA [11] and also a different prediction that the formation of PspA-rich regions of cell membrane may be a dynamic process involving monomeric and other low-order oligomers of PspA [19], as the distribution of PspA stoichiometries fits a four-parameter Burr function, consistent with such a biological mode of action.

It is important to note that the investigators here realized that a single fluorescent spot observed in the far-field imaging regime of a typical fluorescence microscope is not necessarily a true oligomer, as multiple fluorescently tagged monomers in close proximity to each other, separated by less than the optical resolution limit, will appear to be a single spot of fluorescence with roughly the same brightness as the true oligomeric complex. The data here is interpreted in light of *a priori in vitro* data that predict the distribution observed here *in vivo*, lending weight to the model generated from *in vitro* data. The issue of whether observed spots are oligomers or just a collection of monomers for the earlier-mentioned TatA study was dealt with by the researchers using robust probabilistic analysis. Intuitively, also, the fact that stoichiometries were determined for fluorescent spots that were moving makes it unlikely that a loose collection of monomers would move in the same direction at the same speed. In addition, there was supporting molecular-level evidence for a correlation of stoichiometry with diffusion coefficient, producing a reasonable fit from a diffusion model where TatA adopts an open ring configuration, as suggested by single particle electron microscopy, rather than a filled disc. Finally, prior *in vitro* data gave a sufficient reason to expect oligomers of varying size *in vivo*.

In short, an observed spot of fluorescence intensity in any given diffraction-limited image does not necessarily correspond to a single protein, or to a single protein complex. This conclusion can only be made by taking other information into account, such as the fluorescence intensity and so-called ‘point spread function’ spatial extent of each fluorescence spot in comparison with known single fluorophores in the microscope used, the spatio-temporal dynamics of the observed spot and *a priori* expectations from *in vitro* data.

Single molecule imaging has also proved to be an effective tool for the measurement of *in vivo* dynamics of protein complexes. A good example of this has involved seminal studies on the bacterial flagellar rotary motor. These have revealed that molecular stator subunits, composed of a hetero-tetrameric complex of four molecules of the protein MotA and two of the protein MotB, are dynamically exchanged with a membrane pool of freely diffusing stators [12]. Here, the number of such torque-generating units in the motor was shown to be variable but have a mean of approximately 11, in agreement with other studies using non-fluorescence techniques [20,21], and the size of the pool in terms of number of observed MotB molecules in the plasma membrane was measured at approximately 200 molecules per cell. Furthermore, the rate of exchange between the pool and the ring of stator subunits in functional rotary motors was estimated using fluorescence recovery after photobleaching (FRAP) and fluorescence loss in photobleaching (FLIP), to be equivalent to approximately one stator unit every 30 s. This revelation fundamentally altered the way researchers envisioned how the flagellar motor, and perhaps other complex protein-based molecular machines, really functions *in vivo*. Instead of a fully-formed *isolated* system, each motor is in actual fact a *dynamic* structure with components swapping in and out *on the fly*.

In a subsequent investigation by the same research team, another component of the flagellar motor, the protein FliM, which was known to be part of a directional motor switching complex, was shown to similarly swap into and out of the switch complex, dependending on the presence of a key signal factor in the cytoplasm related to the ultimate detection of external chemicals by the cell [22]. How applicable this model is to other protein complexes remains to be seen and will require researchers to take this question up with future single molecule experiments in live cells to investigate with other biological systems, but a recent further example of dynamic turnover of subunits in functional complexes comes from the Lia *et al.* study previously mentioned, where turnover of Pol III in the replisome was observed.

This theme of novel observation of hitherto unknown or unconsidered processes is also illustrated in Lenn *et al.* [23], which suggests a radical re-thinking of oxidative phosphorylation (OXPHOS), the process by which the universal cellular fuel of ATP is generated, on the single cell scale in bacteria. Prior to this study, there has been a major debate with regard to OXPHOS between the solid-state and free diffusion models reviewed in Lenaz & Genova [24]—both of which assumed that the OXPHOS membrane as a whole was structurally and functionally homogenous in the cell membrane. This study demonstrated that the *E. coli* OXPHOS plasma membrane is clearly not structurally homogenous, when observed on the time scale of tens of milliseconds. These observations were qualitatively reported for the bacterium *Bacillus subtilis* [25], but Lenn *et al.* [23], using single molecule detection, were able to quantitatively characterize the heterogeneity of a key OXPHOS enzyme called cytochrome *bd* oxidase in the *E. coli* plasma membrane, and suggest a new working model, the *respirazone hypothesis* [26], for the field of bioenergetics to consider.

## 4. Single molecule cell signalling

In the field of cell signalling, single molecule fluorescence imaging has revealed the *in vivo* dynamics and underlying mechanisms of signal transduction. Both the tyrosine kinase epidermal growth factor receptor (EGFR) [27] and the M1 muscarinic G-protein coupled receptor (GPCR) [13] have been shown to exist as a mixed population of monomers and dimers, with the monomers transiently dimerizing (figure 1*c*). In the case of EGFR, Sako *et al.* [27], were able to directly observe the details of the mechanism of EGFR dimerization that were previously obscure—binding of the EGF ligand to pre-existing EGFR dimers and the transient conformational states of the EGFR dimers. This study was technically the very first published to draw biological conclusions by monitoring single molecules in a live-cell context. Chung *et al.* [28] subsequently showed that ligand binding is not necessary for dimerization of EGFR and that EGFR dimers were primed for ligand binding and signalling. In the case of the M1 muscarinic receptor, by demonstrating reversible dimer formation of this GPCR and no higher oligomeric states, Hern *et al.* [13] suggest a resolution for apparently conflicting data in the literature from ensemble average approaches that previously argued for functional monomers or functional dimers. The dimerization dynamics of other GPCRs have also been reported [29].

By applying single particle tracking and single molecule Förster resonance energy transfer (sometimes popularly described as ‘fluorescence resonance energy transfer’ due to the non-essential prevalence of fluorescence often used, much to the disservice of the eponymous forefather of the technique), or FRET, a very powerful *molecular interaction* technique that monitors the non-radiative transfer of energy between fluorophores with overlapping spectral properties which are within a few nanometres distance of each other, Murakoshi *et al.* [30] provided an insight into signal transduction through the small G-protein Ras. This study was able to suggest that there was binding of Ras proteins to other immobile membrane-associated signalling proteins following GTP activation.

In an interesting variation on the theme of single molecule imaging, while single molecules were not imaged directly, Xu *et al*. [31] nonetheless applied core principles used in the single molecule approach to study insulin signalling with *single molecule sensitivity*. Instead of single molecules, single vesicle fusion events were visualized. By studying the dynamics of these events at the single event level, the investigators constructed probability distributions of vesicle fusion events with respect to vesicle size and time after insulin stimulation, thus proposing three ways in which insulin promotes exocytosis and fusion of glucose transporter-rich vesicles with the plasma membrane of 3T3-L1 adipocytes.

## 5. Endocytosis, exocytosis and synapses

Multi-modal exocytosis has also been reported in human endothelial cells [32] by using single molecule detection. This study, however, reported qualitative observations of two extreme modes, where vesicles release their cargo accompanied by complete fusion with the membrane and where vesicles release their cargo while remaining essentially intact on the membrane surface, and a third intermediate mode. So, while employing the technology to make valuable observations at the single molecule level, this study here did not give the comprehensive, quantitative picture that the single molecule approach generally is able, and aims, to provide.

**Figure 2.**
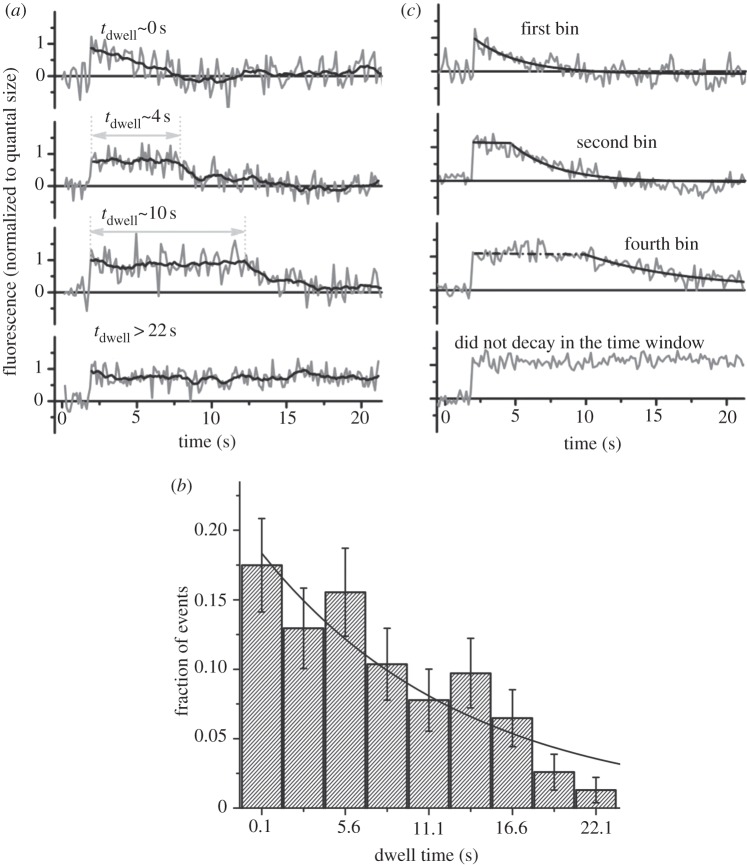
Stochasticity of endocytosis of synaptic vesicles. Vesicles were labelled with pHluorin, a pH-dependent variant of GFP in which quantum yield is dependent on pH (increasing with pH), adapted from [34]. (*a*) Fluorescence traces obtained at individual boutons after single-vesicle exocytosis. Grey lines are raw time traces, and the black line is the running average over 13 points (≈2 s). The endocytic dwell time is defined as *t*_dwell_ = *t*_1/2_ − *τ*_r_*ln*(2), where *t*_1/2_ is the time to decay to 50% of the peak amplitude, and **τ**_r_ is the reacidification time constant (4 s). (*b*) Frequency distribution of dwell times obtained from 150 single-vesicle events obtained from four neurons. Bars are the fraction of events decayed in that time bin, and error bar is the Poissonian noise estimated from the number of events in that bin. All events that did not decay within the observed time (such as the lower trace in *a*) are collected in the final bin (not shown) and included for calculating the fraction of events. The solid line is a fit to a Poisson distribution. The time constant for a Poisson distribution can be estimated from the exponential and gives 13.4 ± 2.4 s as well as reciprocal of the amplitude of the exponential divided by the bin width, which gives 15.0 ± 1.5 s. The fraction of events that remain undecayed within the observation time is in good agreement with that obtained by integrating the exponential fit (17.3%). (*c*) An average of five events from bins centred at 0.1, 2.85 and 8.35 s along with average of five events that did not decay during the observation time window is shown. The solid-grey exponential decays are fits to the reacidification time course. The average time constant from the fit is 3.8 ± 1.8 s.

In contrast, using pH-sensitive GFP, pHluorin [33], as a reporter of synaptic vesicle exocytosis and endocytosis (figure 2), Balaji & Ryan [34] were able to demonstrate that previously observed rapid endocytosis and slow endocytosis events are part of a single continuous population of events at neural synapses. These results indicated that the previously observed rapid endocytosis and slow endocytosis are not distinct processes that are independently regulated but rather are extreme observations of a single stochastic mechanism.

Syx and SNAP-25, collectively called tSNAREs, are involved in synaptic secretory vesicle attachment to the plasma membrane. Knowles *et al.* [35] measured the distribution of these tSNAREs at the single molecule level and suggest that these two proteins form clusters that contain far more tSNAREs than are thought to be required for vesicle attachment, raising the question as to the function of the ‘extra’ tSNAREs. By measuring the diffusive behaviour of these proteins, they were also able to observe multiple diffusive behaviours and quantify the proportion of tSNAREs in each diffusive mode, leading to some speculation about the range of interactions that these proteins have with each other, and with other components of the plasma membrane and the synaptic machinery.

The mobility and distribution of single AMPA glutamate receptors (AMPARs) in and around synapses has also been studied [36]. Here, diffusive behaviours of individual AMPARs were measured and observed to be dependent on whether or not the molecules were within the synapse or in the extra-synaptic region. Three characteristic behaviours were observed in the extra-synaptic region and two were observed within the synapse, and the proportions of each were determined. But also, perhaps most significantly, this study permitted direct visualization of the mechanism by which a previously observed phenomenon, the modulation of AMPAR concentration in the synapse by glutamate, occurs. It was observed that both the proportion of mobile AMPARs and the diffusion coefficient of AMPARs within the synapse increase, while diffusion of extra-synaptic AMPAR does not change upon glutamate stimulation, and that the proportion of AMPARs in the membrane region immediately juxtaposed to the synapse increases in the presence of glutamate. It appears therefore that depletion of AMPARs within the synapse in response to increases in glutamate concentration is due to stimulated diffusion out of the synapse rather than to endocytosis of the receptors, as previously suggested.

## 6. Single molecule gene regulation

It is sensible to probe gene expression at the single molecule level because gene expression is a stochastic rather than a deterministic process [37]—i.e. even though it is possible to say when a gene is *likely* to be expressed, it is impossible to predict *exactly* when it will be in a particular cell; rather, we rely on *probabilistic information* which can really only be obtained by generating such a distribution from single molecule level sampling. Using single molecule live-cell imaging, researchers have uncovered: the mechanisms by which transcription factors find their binding sites [38]; that the process of transcription from constitutive promoters is not solely dependent on RNA polymerase activity [39]; how stochastic binding of transcription factors results in phenotypic switching [40,41] and that final protein levels per cell, using well-characterized bacterial systems, are not necessarily correlated with mRNA levels in an intuitively simple way [42]. Such data are invaluable to computational systems biologists who rely on quantitative results such as these and good *a priori* assumptions.

In eukaryotic cells, single molecule microscopy has been used to probe the structure and dynamics of the nucleus and the import and export mechanisms of the nuclear pore, which is the sole channel of communication between the nucleus and cytoplasm. By observing the motion of single molecules, the nuclear space has been characterized (reviewed in [43]). The transport mechanisms of the nuclear pore have been recently reviewed in [44]. Here, we highlight two recent studies that employ *in vivo* single molecule imaging techniques that challenged expectations and generated a new model for the mechanism of transport through the nuclear pore. Both employ techniques that use the fluorescence intensity spatial distribution profile of single fluorophores in two dimensions, characterized by the point spread function of the microscope in question, to locate fluorophores with approximately 6 nm precision in one case (using bright organic dyes), and with approximately 26 nm precision (using dimmer fluorescent proteins) in the other. The ability to spatially localize sub-cellular components to a nano-scale precision is a characteristic feature of many modern single molecule cellular imaging methods, and is clearly impossible in bulk ensemble approaches.

**Figure 3.**
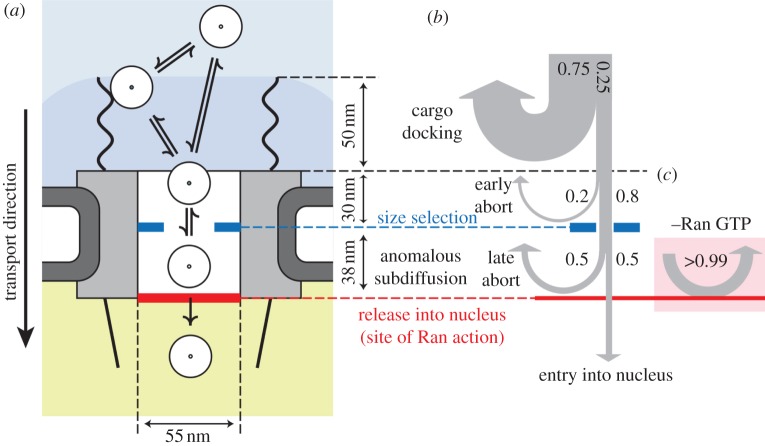
Putting data together—model and summary of results from Lowe *et al.* [45]. (*a*) Cargos arriving from the cytoplasm (white circles) may dock on the cytoplasmic filaments or directly enter the nuclear pore complex (NPC). Once inside the central channel, cargos exhibit anomalous subdiffusion. There is a size selective constriction (blue) within the first 30 nm of the channel. Efficient cargo exit into the nucleus requires Ran (red bar). (*b*) Probabilities of cargos being rejected versus position, highlighting the sequence of steps in cargo translocation. Many cargos interact with the cytoplasmic filaments, but most (75%) immediately rebound into the cytoplasm. The remaining 25% interact with the NPC (grey box) for longer times. Of those, 20% abort early owing to a size gate and 80% reach the central channel. Once inside the channel, in the presence of Ran, 50% of cargos ultimately enter the nucleus and the remaining 50% abort. (*c*) In the absence of Ran, cargos do not enter the nucleus (more than 99% abort) and return to the cytoplasm.

Lowe *et al.* [45] used a permeabilized cell system with quantum-dot-labelled substrates for nuclear import to test various hypotheses of the import mechanism of substrates into the nucleus through the nuclear pore (figure 3). While the cells were not alive in this study, the nuclei studied showed signs of being in a natively functional state. This study quantified the diffusive behaviour of single cargos at nuclear pore complexes, revealing four populations of diffusive behaviour and the proportions of events in each class. The authors were thus able to demonstrate three stages of selectivity in nuclear pore complexes, show that most cargo selection takes place on the cytoplasmic face of the nuclear pore and that the Ran GTPase, a critical factor for directional nuclear import, acts at the nuclear face of the pore.

The export mechanism for messenger RNA, which is synthesized in the nucleus and translated in the cytoplasm, was observed to take place in three temporally distinct steps: docking, transport and release [46]. Contrary to previous expectations, movement through the nuclear pore channel, which was proposed to consist of a hydrogel brush, was not observed to be the rate-limiting step for transport across the nuclear pore. Rather, so-called docking and release steps from the nuclear pore—i.e. entry and exit—were seen to be rate-limiting.

Taken together, these two studies produce a consistent picture of the nuclear pore in which the channel is ‘gated’ at both ends, rather than in the middle, at least with respect to the large molecules which were examined in these studies.

## 7. Single molecule membrane biology

Thus far, this review has focused on the single molecule approach as aiming to measure the distribution of a particular property across a population of molecules to uncover mechanisms that are undetectable by ensemble average measurements. However, a significant contribution has been made to the understanding of living cells by studies that simply tracked the diffusion of single molecules in live cell membranes.

Single particle tracking in live cells could be considered the single molecule version of traditional FRAP and FLIP bulk ensemble measurements as a means of studying diffusion of biological molecules *in vivo.* Spatial resolution of measurement in traditional FRAP/FLIP experiments is diffraction-limited but, as we have seen, in single molecule fluorescence microscopy, it is possible to locate the *intensity centroid* of a single fluorescent dye emitter in a suitably dark background with *ca.* nanometre accuracy for bright organic dyes, and still even only *ca.* tens of nanometres for less photophysically ideal fluorescent proteins. It therefore becomes possible to probe the diffusion length scales below the optical resolution limit and observe putative confinement of molecular mobility on the scale of tens to hundreds of nanometres. This technique was used to measure diffusion in studies already discussed in this review [25,30,35,36,38,45,46].

**Figure 4.**
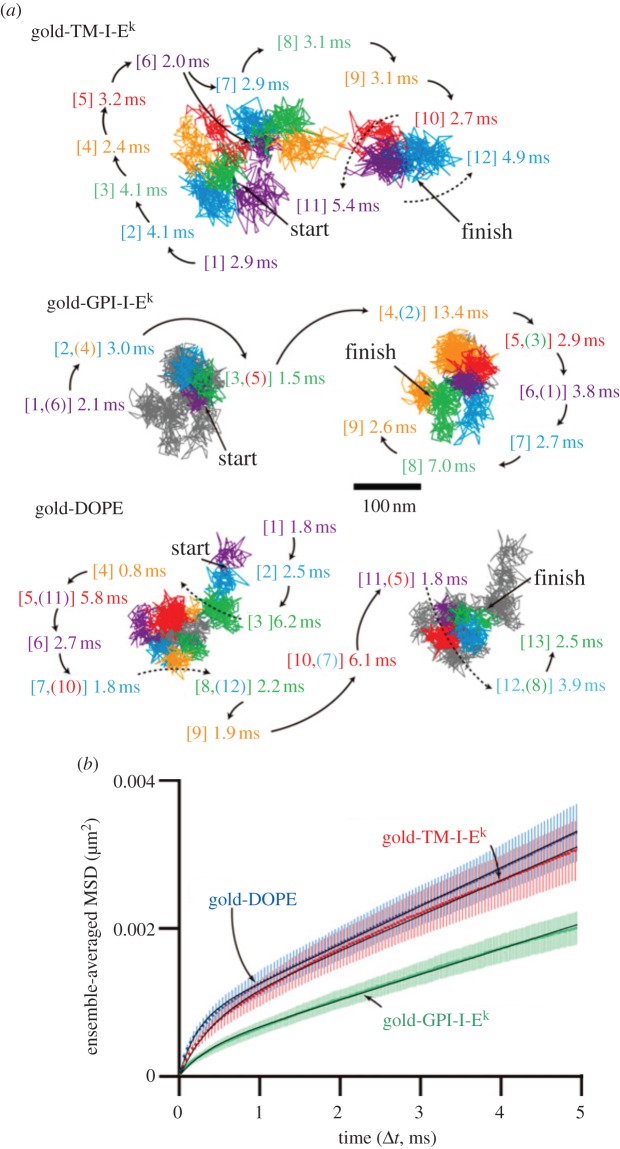
Single particle tracking of membrane components showing comparmentalization of the membranes, adapted from [54]. TM-I-E^k^ (transmembrane protein), GPI-I-E^k^ (raft associated lipid anchored protein) and DOPE (non-raft lipid), tagged with gold particles and observed at a 20 μs resolution, exhibited hop diffusion. (a) Representative 40 ms trajectories (containing 2000 determined coordinates) of TM-I-E^k^, GPI-I-E^k^ and DOPE. Each colour (purple, cyan, green, orange, red, and then back to purple and so on) represents a plausible compartment detected by computer software. The residency time within each compartment is shown and is colour-coordinated with the respective compartment. The numbers in the square brackets indicate the order of the compartments the molecules entered. In the Gold-GPI-I-E^k^ and Gold-DOPE trajectories, due to repeated entrance into the same compartments, the continuous trajectories of GPI-I-E^k^ and DOPE were shown in two separate trajectories placed side-by-side for clarity of viewing, whereas the overall trajectories except for the portions shown in coloured trajectories are displayed in grey lines. When repeated passages across the same compartment took place in these trajectories, the compartment is numbered by two numbers. These results suggest that the compartments move slightly even during 2–20 ms. (*b*) Ensemble-averaged mean square displacement (MSD)-*Δt* plots. MSD of TM-I-E^k^, GPI-I-E^k^ and DOPE tagged with gold particles observed at a 20 μs resolution, averaged over all copies of molecules examined here. Red, TM-I-E^k^; green, GPI-I-E^k^; blue, DOPE.

The cell membrane paradigm has changed [47]. Data from *in vitro* [48], *in silico* [49] and *in vivo* tracking of single molecules in live cell membranes [50–54] (figure 4) converge on the notion that the plasma membranes in eukaryotic cells are compartmentalized fluids [52] rather than a fluid-continuum [55].

Bacterial membrane protein complexes putatively show both free (TatA oligomers [17] and MotB dimers [12]) and confined [25,56] diffusion in the *E. coli* plasma membrane by single particle tracking. These observations suggest that some proteins are affected by diffusion barriers, whereas others are not. It may be the case that the compartmentalized fluid model of biological membranes could apply universally to many different membrane protein systems, and that native membrane structure is the result of complex interactions involving lipid–lipid, lipid–protein, protein–protein and membrane–cytoskeleton interactions. This picture of biological membranes prompts new ways of thinking about membrane processes in general [25], and new questions to answer such as those related to the physical and molecular nature of the observed diffusion boundaries. The functional significance of this new membrane model is also yet to be determined.

## 8. Single molecule virology

Another field in which single particle tracking has proved useful is virology. Single virus tracking has a history going back to the 1980s [57]. Single virus tracking has quantifiably characterized the infection pathway of a number of viruses that use the endocytotic machinery of their host cells for infection. The recruitment of vesicle-forming protein structures to virus binding sites has been quantified [58], along with the dynamics of movement of endocytosed virus particles within cells, demonstrating the movement of virus particles along tubulin microtubles [59–62]. A recent study indicated that the entry dynamics of HIV-1 virions was dependent on the receptors used to initiate endocytocis [63]. The assembly dynamics and release of HIV virions have also been characterized [64–66]. A bacteriophage **λ** search strategy for its entry site has also been proposed [67].

The level of detail in the knowledge about viral infection mechanisms that has been acquired comes from the ability of researches to detect *single* virus particles in living cells, record their movement with high time resolution and skillfully extract the relevant information from the images acquired which, although viruses are clearly not single molecules as such, have used experimental methodologies on living cells with single molecule sensitivity, and have pushed the development of this single molecule cellular biophysics field forward considerably.

## 9. A new frontier: single molecule live-cell super-resolution imaging

The ability to locate single fluorescent molecule tags with precision below the optical resolution limit has formed the basis for several single molecule localization microscopy techniques with too many acronyms to be sensible, including FIONA, FPALM, NALMS, SHREC, SHRImP, STORM, dSTORM, BaLM, PAINT, PALM, TALM and uPAINT. In addition, other ‘patterned illumination’ super-resolution techniques, such STED and SIM, have been very powerful at addressing biological questions, though do not implicitly rely on single molecule detection and are beyond the scope of our review here. However, all of these single molecule localization methods establish a system by which subpopulations of fluorophores within a larger assemblage fluoresce at different times in such a way that at any specific time, fluorescing individuals are ideally separated by distances greater than the optical resolution limit of the system, set by the wavelength of the emitted light and the numerical aperture of the objective lens, and can thus be detected as distinct spots or blurry blobs of fluorescence intensity of typical width approximately 200–300 nm.

In theory, the positions of all the fluorophores in the population can be identified by repeated sampling of the population and these can be plotted to generate a map of fluorophore locations that is limited in spatial resolution by the localization precision of single fluorophores in the sample. In practice, the fluorescence labelling efficiency combined with the real sampling efficiency and the methods of data filtering used (for example, specific methods of noise elimination, thresholding criteria, etc.), and the reconstruction of the spatial location of fluorescent tags must all be taken into account when interpreting results. The technical aspects and methods of super-resolution fluorescence imaging are well reviewed in the literature elsewhere, with a frequency and regularity sometimes so high that one wonders whether anyone has time to do any actual experiments but, with due warning given, we direct the reader to some of these [68–72].

The repeated sampling required for single molecule localization microscopy has typically meant that hundreds or thousands of individual image frames are required to generate an image and, because photon integration time must be sufficiently long to capture the weak fluorescence signal above the noise, the effective temporal resolution is typically very low—seconds to minutes—in comparison with most molecular-scale events. Most experiments thus far have therefore been done on fixed samples (not within the scope of our review here). There are a growing number of reports of super-resolution imaging of live-cell samples. However, the number of studies that use these techniques to directly address biologically relevant questions are surprisingly few as most of these studies have focused on demonstrating various strategies to improve temporal resolution of image capture, and that these relatively new techniques actually work on more or less live samples [73–76]. In general, what has been constructively generated are images of structurally well-characterized systems and slow-moving structures, where a temporal resolution of seconds is sufficient, and while these proofs-of-principle are significant in themselves as establishing tools for cell biology, most have neither probed systems where sub-diffraction distances between molecules address deep biological questions in the field nor raised new questions.

There is also a significant issue in many of the reported ‘live-cell’ super-resolution studies not being definitively performed on ‘living cells’ *per se*. The problem here is that many of the super-resolution techniques either use relatively short wavelength ‘long uv’ laser excitation to activate and/or photoswitch fluorescent proteins or organic dye tags, as well as requiring relatively long total imaging times to perform full image reconstructions. The combination of the two results in potentially very significant photodamage of cellular content from the accumulation of a free-radical pool, implying a danger of creating artefactual sub-cellular features that do no exist in the native unperturbed cell, as well as damaging the cell irreversibly to the point of killing it.

In other words, although cells may be living at the start of a super-resolution experiment, one must be suitably cynical as to whether they are alive at the end of it, unless a suitable cell viability/functionality assay can be performed on each same cell both before and after. Many leading researchers in the super-resolution bio-imaging community have become a little blasé to this problem, however highly sensitive single cell functionality assays do exist which could be used were efforts to be made. For example, one such exists for bacterial studies since it involves measuring the speed of the bacterial flagaller motor using a high time resolution non-fluorescence laser interferometry method [20]. Flagellar motor speed is known to be a highly accurate indicator of protonmotive force, which in turn is known to be very sensitive to the presence of free-radicals in the cell.

Here, we discuss just a few live-cell studies where the investigators used the super-resolution images obtained by single molecule localization to address specific biological questions. The first is work by Fu *et al.* [77] on the bacterial division protein FtsZ. The researchers here used high-intensity excitation and high-imaging frame rates to record PALM images of the protein FtsZ in live *E. coli* within 20 s, and successfully showed that images obtained from fixed samples in this case are similar to those from live cells. The quality of the images shown in the paper is not high for the purposes of the study, which was to image the FtsZ ‘ring’ observed in diffraction-limited microscopy at high time resolution in order to distinguish between molecular models of its structure and assembly. The investigators did observe single lines that might be expected for a single closed ring structure and in addition saw patterns that were invisible to other imaging techniques. However, their argument that these additional patterns represent a variety of compressed helices of varying pitches is not strong. The researchers validated these claims by comparing the raw PALM images to simulated data from assumed FtsZ configurations based on ‘visual inspection of the images’, which makes their logic uncomfortably circular.

The approach of comparing ‘real’ to ‘simulated’ data is valuable but would have been substantially more convincing in this case if the investigators had presented a quantitative measure of how good the match was between the simulated and real images, with a comparison of ‘goodness-of-match’ between simulated images using helix and other possible models—for instance, a series of unconnected closed hoops, which may or may not touch, of variable pitch. The argument that the images with multiple bands actually represent helices of variable pitch would be significantly strengthened if the data were thus demonstrated to match this ‘slinky-spring’ model the best in comparison with other models. In an unrelated line of reasoning, the researchers also argued that their measurements of the ‘band’ width (approx. 110 nm), and the observation that this apparent width does not change with increasing FtsZ concentration, supports a loose bundle model of FtsZ arrangement in the division ring, rather than previously proposed flat ribbon and random ribbon models. Admirably, the authors identified a biological problem of significant interest for which single molecule localization super-resolution imaging is the ideal tool in theory. However, the success of the approach in this instance is brought into question by the quality of the data obtained.

This example is not unique in the field of single molecule cellular biophysics research in this regard. If anything, the case study exemplifies some of the typical potential limitations in interpretation when, as is very often the case, the effective signal-to-noise ratio for such experiments is often very low, in the range of approximately 1–10, with the most pioneering studies in general being the most speculative for which the signal-to-noise ratio may be sometimes only marginally above 1. In other words, working right at the technological cusp of single molecule detection for events which are only just experimentally observable above the level of noise.

More convincing results were obtained by Hess *et al.* [78] who used the super-resolution technique of FPALM on living fibroblast cells to obtain a map of haemagglutinin. Haemagglutinin is a membrane protein associated with cholesterol-rich lipid domains, also popularly referred to as *lipid rafts*, which are characterized by a typical length scale of around approximately 10–200 nm. Importantly, the elongated shapes of the observed haemagglutinin clusters and their irregular boundaries observed have been considered as evidence to support the notion that certain membrane *nano-domains* may be generated by membrane–cytoskeleton interactions rather than purely by lipid phase-transition behaviour.

More recently, single particle tracking and PALM imaging have been combined in a technique termed sptPALM or TALM [79] allowing the ‘simultaneous’ tracking of large (more than 1000) assemblages of molecules in a single live cell, rather than the few (less than 10) molecules that can be reliably distinguished and tracked in a single sample in the diffraction-limited studies discussed earlier. This technique has thus far only been applied to study the dynamics of membrane proteins. It speeds up the data collection process and allows for a more comprehensive view of cell membranes at the single cell level, opening the door for researchers to study cell-to-cell variation in membrane structure. The image integration times used approached video-rate with approximately 50 ms per image frame and were suitable for the dynamics of the system studied. Such frame rates were achieved by intense irradiation and the signal-to-noise ratio enhancement provided by the total internal reflection imaging configuration used. The single and most recent two-colour sptPALM/TALM studies [80] published by Lippincott-Schwartz and co-workers represent the first comprehensive diffusion maps of membrane proteins determined directly at a single cell level and add detail to the new membrane paradigm discussed already. Consistent with observations discussed earlier, these studies observe distinct diffusive behaviours of different protein populations, a variety of diffusive behaviours within populations and varying frequency of confinement and co-localization with other membrane proteins. As reflected in the published research papers themselves, the data that these images have generated is immense and has clearly not been interrogated as fully as is possible; it is clear that there is significant potential to analyse them in many more ways to probe more questions than those addressed in these papers directly, that rather serve principally as technical proofs-of-principle.

Obtaining images of cells with high spatial resolution without structural artefacts due to fixation—either by chemical or cryogenic means—remains an important motivation to pursue live-cell super-resolution imaging. However, perhaps the unique advantage that super-resolution live-cell imaging promises will be to uncover dynamic processes at unprecedented levels of spatial detail where the live-cell behaviour of all the individuals in populations of molecules, rather than a sub-sample, can be visualized within a short time frame—i.e. effectively ‘simultaneously’. As the research community works towards seeing the *molecular clockwork* inside cells in full action, the volume of data generated will require significant investment in resources dedicated to data management and automated analysis in order for the data to be useful and interpreted in a timely manner.

## 10. Summary and conclusion

Live-cell single molecule experimentation, primarily through fluorescence microscopy techniques, has revealed details of processes and structures in living cells by direct observation of single molecules. It has proved particularly useful for determining *in vivo* stoichiometries of protein complexes and assemblies, and for characterizing the movement of molecules in cells and on cell surfaces. It also provides a tool for quantifying stochastic processes and has the sensitivity to observe small fluctuations that have profound effects at the cellular level. In some cases, solutions for controversies in the field have been provided, while in other cases novel observations have suggested new paradigms. In all cases, new tools for quantitative *fine-grain* measurement have been provided for hypothesis testing and a wealth of data is now potentially available for *in silico* modelling.

While the work still remains a significant technical challenge, it is a fruitful nexus for biologists, physicists, chemists, computer scientists, mathematicians and engineers, where, in effect, new technology and analytical approaches have the potential to answer some very old biological questions, and provide new questions to answer, and where questions from one field can drive technology development in other fields.

If living things are the sum of molecular interactions, as high-quality single molecule live-cell data accumulate, then the potential to make connections between the parts and the whole increases. This is ultimately a reductionist approach to the study of life and while it is certainly true that biological systems in general do have *emergent* properties, where the parts do not fully explain the whole, it cannot hurt to find out how and where the useful boundaries between these two approaches lie.
